# 

**Published:** 1916-10

**Authors:** 


					﻿Vol. XXXII
OCTOBER, 1916
No. 4
TEXAS
Medical ..^nal
ESTABLISHED JULY, 1885
PUBLISHED MONTHLY AT AUSTIN. TEXAS. BY
MRS. F. E. DANIEL, -	Publisher and Managing Editor
Subscription, Si.00 a Tear, tn Advance. -	.	.	. Single Coples,10 Cents
Entered at the Postofflce at Austin, Texes, as second class mail matter.
OS
<
a
>
q'
Z
MM '
E
r
				

